# Gastrointestinal stromal tumour of the rectum and intestinal obstruction: case report

**DOI:** 10.3332/ecancer.2020.1139

**Published:** 2020-11-10

**Authors:** Ana Karla Uribe Rivera, Andrés Guevara Jabiles, Ivan Chavez Passiuri, Elica Garcia Leon, Melvy Guerrero Quiroga, Renier Cruz Baca, Jossue Espinoza Figueroa, Nelson Purizaca Rosillo, Eduardo Payet Meza

**Affiliations:** 1 Surgical Oncology Resident at National Cancer Institute (INEN), Lima 34, Peru; 2 Department of Abdominal Surgery at National Cancer Institute (INEN), Lima 34, Peru; 3 Department of Medical Oncology at Regional Hospital of Lambayeque, Lambayeque, Peru; 4 Department of Oncological Pathology at National Cancer Institute (INEN), Lima 34, Peru; 5 Fellow of Oncological Pathology at National Cancer Institute (INEN), Lima 34, Peru; 6 Radiology Resident at National Cancer Institute (INEN), Lima 34, Peru; 7 Clinical Genetic Resident at National Cancer Institute (INEN), Lima 34, Peru

**Keywords:** case report, rectal gastrointestinal stromal tumour (GIST), CD117, DOC-1, C-KIT mutation, exon 9

## Abstract

We report the case of a 75-year-old female patient with a big tumour in the lower rectum with intestinal obstruction and lower gastrointestinal bleeding history who underwent a tumour biopsy under laparotomy and end colostomy at another hospital in Peru. She came to our institution for clinical evaluation with a pathology result of a rectal gastrointestinal stromal tumour. An extra elevator abdominoperineal resection was performed with tumour-free margins. The histology confirmed a high-grade (G2) rectal gastrointestinal stromal tumour with a mitotic index of 27/50. DOC-1 (+) and CD117 (+) in immunohistochemistry. Genomic DNA was extracted from the paraffin-fixed tumour sample, and c.1504_1509dupGCCTAT (p.Ala502_Tyr503dup) mutation was detected in exon 9 of the KIT gene. Imatinib 400 mg per day for 3 years was indicated as adjuvant treatment. Currently, she has a disease-free survival of 12 months.

## Introduction

The gastrointestinal stromal tumour (GIST) term was established by Mazur and Clark in 1983, in reference to non-epithelial tumours of the gastrointestinal tract without leiomyosarcoma features [1] arising from the interstitial cell of Cajal or its precursor with malignant tendencies. This rare mesenchymal tumour accounts only for 1%–3% of all gastrointestinal malignancies and is predominantly located in the stomach (60%–70%). GIST in the rectum is extremely rare and represents less than 5% of all gastrointestinal stromal tumour [2] and 0.1% of all colorectal tumours with an estimated incidence rate of 0.45 persons per million [3, 4]. The median age is around 60–65 years old [5], with a similar clinical presentation to rectal adenocarcinoma (rectal bleeding, constipation, abdominal and pelvic discomfort). Intestinal obstruction due to rectal GIST is uncommon.

## Case report

**Figure 1.**
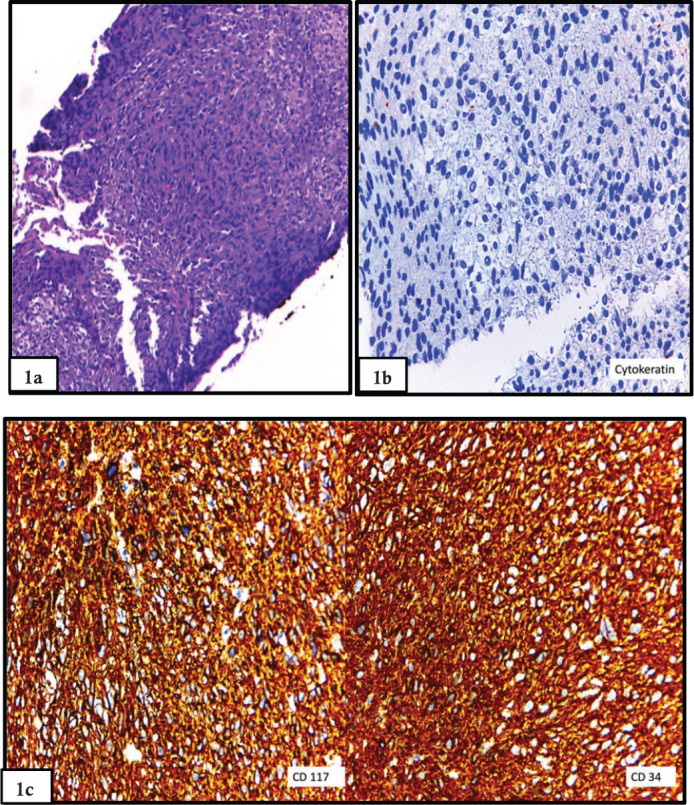
(a): Biopsy prior to abdominoperineal resection showed a spindle cell proliferation, with bland nuclei and eosinophilic cytoplasm and inconspicuous nucleoli (haematoxylin and eosin stain). (b): Biopsy prior to abdominoperineal resection showed a spindle cell proliferation, with bland nuclei and eosinophilic cytoplasm and inconspicuous nucleoli. The stain for Cytokeratin AE1/AE3 was negative, which favoured the mesenchymal nature of the neoplasm. (c): Staining for CD117 and CD34 was diffusely positive, thus favouring a gastrointestinal stromal tumour. Confirmation with DOG-1 was not performed in the biopsy.

**Figure 2.**
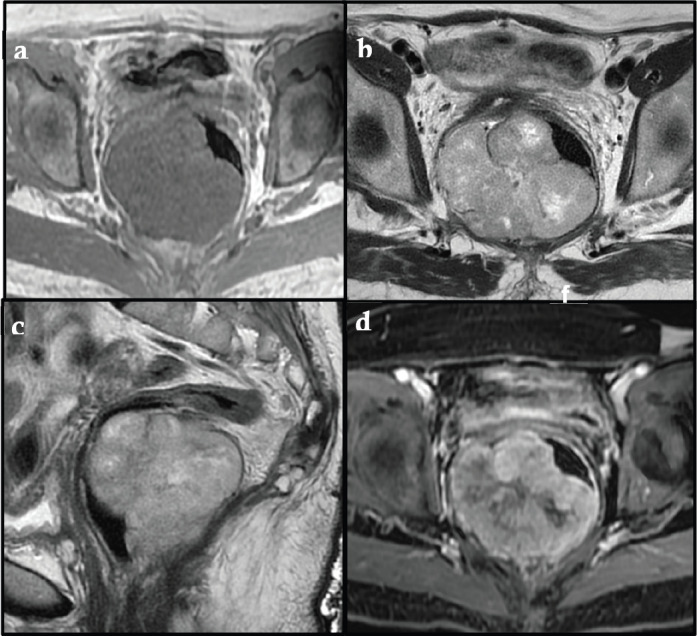
(a, b, c): Axial T1-weighted MR and Axial, Sagittal and Coronal T2-weighted MR respectively showed a large isointense mass in T1, iso-hyperintense in T2, with well-defined edges located in the pelvic region. The posterior and right lateral walls of the median and lower rectum were involved, attached to the mesorectum and levator ani muscle with a predominantly exophytic component and an intraluminal component. MR showed a tumour mass compressing and displacing the rectum to the left and the uterus superiorly. No lymphadenopathy was observed. (d). Post-contrast Axial Fat-Suppressed T1-weighted MR shows a solid component of the mass-enhanced heterogeneously.

This is about a 75-year-old Peruvian female with 4 months of abdominal pain associated with low gastrointestinal bleeding and faecal incontinence who had a laparotomy with tumour incisional biopsy and end colostomy at another hospital. She was referred to the National Cancer Institute with a pathology report of rectal GIST (Figure 1). A rectal tumour at 1 cm from the anal verge at digital examination occupying 80% of circumference without anal sphincter tonicity was found. An extensive solid exophytic lesion of 7.6 × 9.3 cm with large necrotic areas restricting diffusion was shown on MRI. The tumour was attached to the mesorectum and levator ani muscle with unsafe and positive circumferential resection margin (+CRM). No metastatic disease was found (Figure 2).

**Figure 3.**
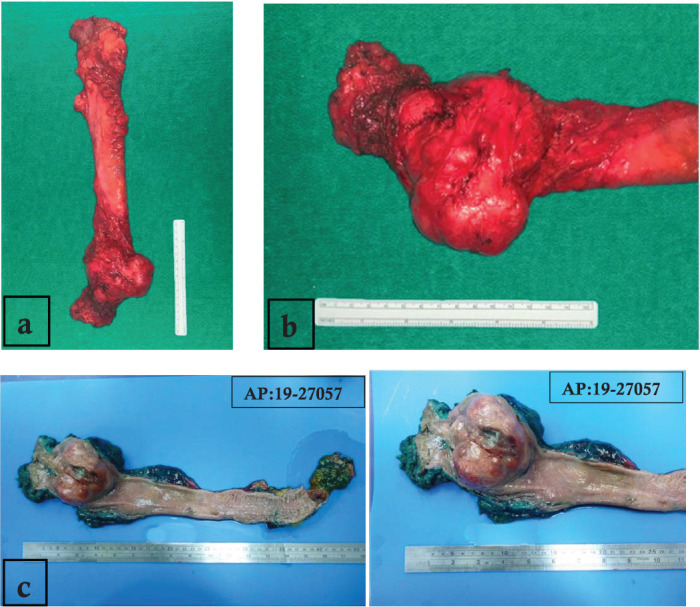
(a): Surgical specimen of extra elevator abdominoperineal resection. (b): Tumour on lower rectum with well-defined edges and predominantly exophytic component. (c): Macroscopically, the tumour was a fleshy, tannish-brown, multilobular, well-circumscribed mass of 8.0 × 7.0 × 6.5 cm, centred in the muscularis propria of the rectum with ulcerated mucosa.

An extra elevator abdominoperineal resection of the primary tumour was performed on May 2019. Pelvic examination under anaesthesia showed a tumour at 3 cm from the anal verge with a wide base depending on the right and posterior walls of the rectum (Figure 3).

**Figure 4.**
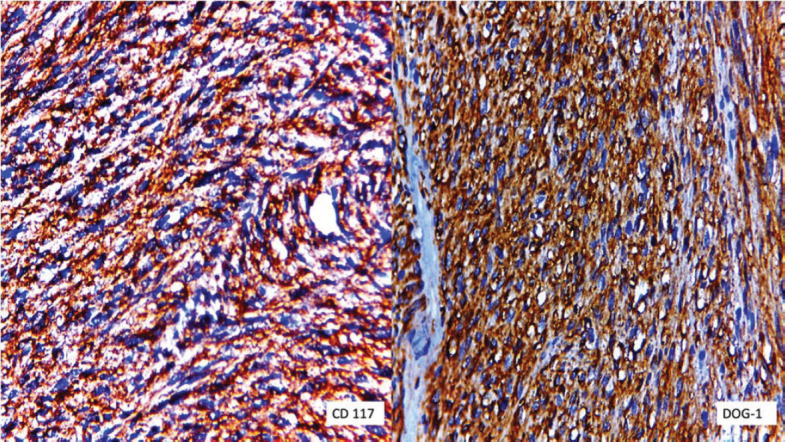
Immunohistochemical staining for CD117 was positive and specific DOG-1 was diffusely positive.

The final pathology reported a tumour of 8 × 7 cm, distal margin of 4.5 cm and proximal margin of 30 cm, tumour-free margins. The histology confirmed a fusocelular subtype of rectal GIST with a mitotic index of 27/50, G2 and 30% of necrosis. No lymph node metastasis (0/53) was found. The final pathologic stage was pT3N0. Immunohistochemistry showed DOC-1 (+) and CD117 (+) (Figure 4).

The patient began adjuvant treatment with Imatinib 400 mg per day for 10 weeks after surgery. Until the date of her last follow-up, there was no evidence of recurrent disease by imaging studies (12 months).

Genomic DNA was extracted from the paraffin-fixed tumour specimen, and a Sanger sequencing reaction was performed from exons 9, 11, 13 and 17 of the KIT gene and exons 12, 14 and 18 of the PDGFRA gene. The mutation c.1504_1509dupGCCTAT (p.Ala502_Tyr503dup) was detected in exon 9 of the KIT gene.

## Discussion

Rectal GIST is a rare condition, which is characterized by a large tumour mass with well-defined margins. Extraluminal location with an epicentre located outside the rectum is usually found in most cases and described as an adverse prognostic factor, regardless of the tumour size [6]. In our country, the prevalence of gastrointestinal stromal tumour of the rectum is less than 4% of all GIST tumours [7, 8].

MRI helps to determine the origin of the tumour and allows an adequate assessment of the surgical pelvic floor, the involvement of adjacent organs and CRM. T2-hyperintense foci with heterogeneous pattern of enhancement and T1-isointense image as skeletal muscle are frequently seen as in our case. Calcifications and areas of haemorrhagic foci can be present [9, 10]. The presence of intramural degeneration signs with cystic changes, haemorrhage and calcification should also exclude the possibility of lymphoma [6].

A biopsy is essential, ideally, with a transrectal core biopsy. Assessment of tumour behaviour by molecular analysis, especially in large tumours that require radical surgery or neoadjuvant/adjuvant treatment [11], and patient referral to a specialist centre for multimodal management should be done. Around 90% of GISTs are related to gain-of-function mutations in KIT and PDGFRA genes [12, 13]. Duplication was found in exon 9 of the KIT gene in our patient. This is the second most frequent mutation in GIST [14] and is related to primary non-gastric GIST in small bowel and rectum [15]. GIST appears sporadically in most cases, and some are related to hereditary syndromes such as Neurofibromatosis type 1 or Carney–Stratakis Syndrome [16]. Wild-type GISTs (WT-GIST) have no gene mutation, and treatment failure with tyrosine kinase inhibitors is shown. Mutational analysis must be performed for therapeutic management and survival improvement according to the imatinib dose [17].

A well-circumscribed mass of highly variable size (from <1.0 mm to > 20.0 cm) is usually shown macroscopically. The cut surface may show foci of haemorrhage, cystic change or necrosis in large lesions [18]. Tumours consist of uniform spindle cells or epithelioid cells arranged in lobules microscopically. Nuclear pleomorphism is rare, the cytoplasm is eosinophilic and cytoplasmic vacuoles are common. Vessels are typically inconspicuous, and there might be a myxoid or myxochondroid background. ‘Skeinoid’ fibres, which are coarse, wire-like collagen bundles, are present in small bowel tumours.

Invasion into the mucosal and tumour necrosis are poor prognostic factors. The so-called ‘paediatric-type’, which is an SDH-deficient GIST with plexiform growth, commonly epithelioid with lymph node metastasis [19], is not found in the present case. Small intestinal and colonic GIST usually have spindle cell morphology, with diffuse sheets or vague storiform arrangement of cells, and most frequently present a feature spindle cell morphology [18].

Histologic grading is important in soft tissue sarcoma staging but not well suited for GIST. They usually have aggressive features despite having low mitotic rates [20]. The grade is determined entirely by the mitotic activity according to The College of American Pathologists (CAP): GX: grade cannot be assessed; G1: low grade, mitotic rate ≤5/5 mm2; and G2: high grade, mitotic rate >5/5 mm2. The CAP adopted the criteria for risk stratification of Miettinen and Lasota and included anatomic site as a factor [21, 22]. Most GISTs have kit mutations and are CD117/c-kit stain positive (95%) at immunohistochemistry, and about 70% of GISTs express CD34. DOG-1 is an antibody discovered using gene expression profiling and is also expressed by most GISTs (>99%). Most KIT negative/DOG1 positive GISTs are gastric or extra visceral GISTs and harbour a PDGFRA mutation [23, 24].

Following the guidelines for risk assessment of primary GIST [19, 20], we classified it as a high-grade GIST for the risk of progressive diseases, due to the rectal location and a mitotic index >5 per 5 mm^2^.

Surgical resection is the treatment for all resectable rectal GIST patients [17, 25]. The anatomical relationship of the rectum in the pelvis, the sphincter complexity, risk of tumour rupture and positive surgical margins makes a challenging management condition. Technically, a total mesorectal excision (TME) could be the best approach, but it is not essential oncologically, as lymph nodes are usually not involved. Only 10% of all GISTs present metastatic lymph node disease and peritoneal seeding. Malignant stromal tumours are usually associated with tumour rupture.

Local tumour resection (LTR) or sphincter-preserving procedures can be performed for small or low-grade tumours [26]. Different conservative approaches such as transsacral (Kraske) for posterior low-rectal GIST and transvaginal or transperineal approach in women with smaller tumours on the anterior rectal wall have been described [27].

Before imatinib, surgical procedures were more radical and extensive for large and high-risk tumours with aggressive features. Low anterior resection or an abdominoperineal resection are still indicated for rectal tumours located below 5 cm from the anal verge with a high risk of positive CRM [28]. Some authors always recommended both surgical approaches depending on the tumour size (>2 cm) and the distance from the dentate line [29].

With the introduction of imatinib in the management of GIST, some absolute indications for surgery have changed. The benefit of a previous pathological study supports the use of neoadjuvant treatment in some cases even for dose resectable rectal GIST. The neoadjuvant treatment with imatinib is used when R0 surgery is not feasible or when a function-sparing surgery is planned [17]. Wilkinson * et al.* [11] reported 13 patients who had different surgical procedures: nine of them received neoadjuvant imatinib and seven had a downsizing tumour allowing sphincter-preserving procedures.

Gastrointestinal stromal tumour of the rectum with intestinal obstruction emergency is a very rare situation. In this case, a diverting colostomy with a biopsy of the tumour was performed at a general hospital. An incisional biopsy is considered as a tumour rupture according to the Oslo criteria [30] and can overtake a high risk of recurrence after complete resection of the primary tumour [31, 32].

Advantages reported for neoadjuvant treatment are tumour downsizing, mitotic activity reduction, high rates of organ and sphincter preservation, low rates of positive margins, low recurrence and less morbidity in patients with high-risk GIST tumours [28, 33]; however, there is no evidence of such benefits for patients with previous incisional biopsy.

Disease-free survival (DFS) depends on the risk stratification. Better results are achieved in patients with intermediate and high-risk tumours [34]. Actually, the largest single-institution experience reported by Cavnar * et al.* [33] showed 91%—5-year overall survival, 100%—disease-specific survival (DSS) and 82%—recurrence-free survival significantly higher in the imatinib era with no local recurrences. Similar results have been reported in other studies with higher rates of R0 resections [27].

There are no differences in recurrence-free survival (RFS) and overall survival (OS) among LTR or more radical resections. The decision on the optimal surgical treatment mainly depends on the tumour size, mutational status and the surgeon’s capacity [35]. The lack of evidence in large-scale prospective studies makes neoadjuvant treatment, surgical approach and prognosis for rectal GIST still controversial for some authors [25, 28]. In the present case, a radical approach was performed; the size of the tumour in the lower rectum and the high risk of recurrence due to previous incisional biopsy through the mesorectum, with the intraluminal ulcerated component (Figure 3a and b), were the most important considerations for the management decision.

## Conclusion

Optimal management of gastrointestinal stromal tumours of the rectal is challenging.

A biopsy is essential for the molecular assessment by fine-needle aspiration cytology (FNAC) or trans rectal core biopsy.

Multimodal treatment for GIST of the rectum should be managed in a specialized centre.

Incisional biopsy must be avoided since tumour rupture and seeding could raise the risk of recurrence. In such cases, a more radical surgical procedure must be considered.

The approach and surgical procedure with tumour-free resection margin have no significant impact on prognosis and survival.

## Conflicts of interest

We know of no conflicts of interest associated with this publication.

## Funding statement

There has been no significant financial support for this work that could have influenced its outcome.
